# Advancing clinical integration in undergraduate dental education through AI-enabled instructional design: a qualitative thematic analysis

**DOI:** 10.1080/10872981.2026.2750709

**Published:** 2026-10-19

**Authors:** Selçuk Akturan, Ömer Faruk Güdük, İpek Nur Berberoğlu, Elif Özlem Ardıç Kemerkaya, Salman Yousuf Guraya

**Affiliations:** a Faculty of Medicine, Department of Medical Education, Karadeniz Technical University, Trabzon, Türkiye; b Department of Pediatric Dentistry, Faculty of Dentistry, Karadeniz Technical University, Trabzon, Turkey; c Department of Educational Sciences, Division of Educational Measurement and Evaluation, Trabzon University, Trabzon, Türkiye; d Gulf Medical University College of Medicine, Ajman, United Arab Emirates

**Keywords:** Artificial intelligence, dental education, outcome-based education, clinical integration, curriculum development

## Abstract

**Background:**

Despite growing interest in artificial intelligence (AI) in health professions education, qualitative evidence for integarting AI-supported instructional redesign on teaching practices and students' experiences in undergraduate dental education remains limited. The aim of this study was to explore the educational impact of AI-supported instructional redesign of lessons in undergraduate dental education.

**Methods:**

We conducted this inductive qualitative thematic analysis in an undergraduate dental education setting. Existing lessons were redesigned using AI-supported recommendations to strengthen alignment between learning objectives, authentic clinical contexts, and expected clinical outcomes. Data were collected through classroom observations and students’ focus group discussions. An inductive thematic analysis was performed, and findings from multiple data sources were curated, collecetd, and triangulated to enhance credibility.

**Results:**

AI-supported instructional redesign transformed teaching by promoting authentic clinical scenarios, clinically oriented discussions, and explicit reasoning activities. Thematic analysis identified four interconnected themes: (1) a shift from teacher-centred knowledge delivery to facilitation of clinical reasoning; (2) transformation from passive attendance to active cognitive engagement; (3) building clinical meaning through authentic clinical contexts and collaborative dialogue; and (4) creating conditions for professional learning through psychological safety. AI functioned as a pedagogical design-support tool rather than replacing the educators’ instructional role. Overall, the observed educational changes appeared to arise primarily from the pedagogical redesign enabled by AI-supported planning rather than from students' direct interaction with AI.

**Conclusion:**

This study endorses the role of AI-supported instructional redesign in enriching the existing undergraduate dental curriculum. This approach can potentially help educators align learning objectives, instructional strategies, and authentic clinical contexts. The reported educational modifications reflect primarily the implementation of learner-centred and clinically oriented pedagogical strategies, with AI functioning as a design-support mechanism rather than as a direct instructional agent.

## Article highlights

- AI supported educators in redesigning clinically oriented teaching.
- AI improved alignment between learning objectives and clinical outcomes.
- AI-supported teaching promoted active clinical reasoning.
- Authentic clinical contexts enhanced meaningful learning and knowledge transfer.
- AI-supported instructional redesign was feasible and educationally valuable.

## Introduction

One of the most prominent trends in contemporary health professions education (HPE) is the design of teaching and learning processes that are learner-centered, clinically relevant, and capable of fostering transferable competencies. Dental education, in particular, requires a close integration of theoretical knowledge with clinical practice. However, preclinical lessons are frequently perceived by students as disconnected from authentic clinical contexts, which may reduce learning motivation and hinder the transfer of knowledge to future clinical practice [1,2].

Clinical outcome-oriented educational approaches advocate that learning objectives should extend beyond the acquisition of cognitive knowledge to encompass higher-order competencies such as clinical reasoning, decision-making, patient safety, and professional attitudes [3]. This perspective aligns closely with the principles of outcome-based education (OBE), which emphasizes the alignment of learning outcomes, instructional strategies, and assessment with the competencies required in real clinical practice [4].

Despite the increasing emphasis on outcome-oriented education, an important challenge remains: to what extent are existing lessons meaningfully connected to intended clinical outcomes, and how can educators systematically achieve this integration? In many educational settings, lesson design continues to rely largely on individual teaching experience, resulting in varying degrees of explicit integration between theoretical content and clinical practice. Consequently, clinical outcome orientation is often implicit rather than intentionally embedded within instructional design [5]. These challenges highlight the need for practical, flexible, and pedagogically informed tools that can support educators in redesigning existing lessons.

Recent advances in artificial intelligence (AI), particularly large language models such as ChatGPT, have introduced new opportunities for educational design in HPE. Emerging evidence suggests that these tools can assist educators in curriculum development, refinement of learning objectives, generation of authentic clinical scenarios, and creation of interactive learning materials [6,7]. When appropriately designed and guided, ChatGPT has the potential to support educators by aligning learning objectives with clinical competencies, enriching lesson content with clinically relevant examples, and generating discussion prompts and reflective questions that promote active student engagement [8].

However, despite the growing interest in AI-assisted education, limited research has examined how ChatGPT can be integrated as an educator support tool within existing curricula, particularly for enhancing the clinical relevance of preclinical teaching [9–11]. In dental education, existing GenAI research has predominantly focused on student- or clinician-facing applications, particularly evaluations of chatbot performance in licensing examinations, discipline-specific questions, and responses to clinical queries [12–15]. Comparatively little attention has been given to the use of GenAI as a faculty-facing instructional design tool for systematically redesigning existing teaching. Qualitative evidence examining how such AI-supported redesign is enacted in the classroom and experienced by students also remains limited. This gap is particularly evident in dental education, where few studies have proposed or evaluated systematic models for using ChatGPT to enrich existing lessons with clinically meaningful learning outcomes.

This study aimed to explore AI-supported instructional redesign in undergraduate dental education by examining modifications in clinical outcome-oriented teaching practices through classroom observations, examining how AI-generated instructional recommendations contributed to the redesign of lessons, and by exploring students’ perceptions and experiences of the redesigned learning environment.

## Materials and methods

### Study design

This study employed a qualitative descriptive case study design to explore the feasibility and perceived value of integrating a AI-supported instructional approach into existing undergraduate dental lessons Guided by an interpretivist paradigm, the study sought to understand how educators and students experienced the enhancement of preclinical teaching through clinically oriented instructional design REF. We adopted a qualitative case study as this approach enables an in-depth exploration of complex educational phenomena within their real-world context while incorporating multiple sources of evidence [16,17].

### Study setting and participants

The study was conducted at the Karadeniz Technical University Faculty of Dentistry and involved fourth-year undergraduate dental students and faculty members. The study focused on two core preclinical lessons within the fourth-year undergraduate dental curriculum: *Atraumatic Restorative Treatment and Contemporary Approaches* and *Abscess Management and Gangrene in Children*. Each lesson was delivered as a 45-minute face-to-face teaching session.

At the time of the study, the undergraduate dental curriculum did not include formal instruction on the educational use of generative artificial intelligence (AI). Students had not received structured training in the educational application of ChatGPT or other generative AI tools. In the present study, AI was used exclusively to support instructional redesign by faculty members during lesson planning. Students did not directly interact with AI during the educational intervention.

A purposive criterion sampling strategy was employed. Students were eligible if they were actively enrolled in the fourth year of the undergraduate dental curriculum during the study period. The baseline (routine) lessons were delivered as part of the regular curriculum and were attended by 86 students for *Atraumatic Restorative Treatment and Contemporary Approaches* and 92 students for *Abscess Management and Gangrene in Children*.

Following AI-supported instructional redesign, students were verbally invited by the research team to attend the redesigned lessons. Participation in the redesigned sessions was entirely voluntary, and students were informed that participation or non-participation would not influence their academic evaluation. Consequently, 80 students attended the redesigned *Atraumatic Restorative Treatment and Contemporary Approaches* lesson and 76 students attended the redesigned *Abscess Management and Gangrene in Children* lesson.

The two lessons were delivered by two faculty members from the same academic department. Both instructors had comparable experience in undergraduate dental education and had previously completed formal faculty development training in educational methods, providing a relatively homogeneous instructional background while allowing exploration of instructional changes following AI-supported lesson redesign.

Following completion of the redesigned lessons, students who had experienced both the routine and the AI-supported instructional approaches were invited to participate voluntarily in focus group discussions to compare their learning experiences across the two teaching approaches.

### Data collection

Initially, the two lessons were observed in theirconventional educational setting using a structured observation guide developed through a review of the literature on classroom observations and clinical teaching, and refined by the research team to align with the objectives of our study (Supplementary Material 1). The observation guide comprised seven sections: general information and six observational domains covering learning outcomes and instructional alignment, learner engagement and interaction, instructor’s role, teaching methods and learning activities, feedback and assessment, and reflection and session closure. Across the six observational domains, 19 criteria were marked as No (0), Partially (1), or Yes (2). These numerical labels were used solely as structured anchors to facilitate observers’ criterion-level judgments and overall qualitative evaluation; total scores were not calculated or used for quantitative analysis or comparison between sessions. Each domain also included space for qualitative field notes, which constituted the primary observational data used in the qualitative analysis. The same structured observation guide and observation procedures were subsequently used to observe the redesigned sessions.

Each lesson was observed once in its entirety by at least two members of the research team, who served as non-participant observers and did not interfere with the teaching process. The same group participated in the observation of the conventional and redesigned sessions. The observers were selected from the research team based on their experience in HPE research and their familiarity with qualitative classroom observation. Before the data collection, the research team reviewed the observation guide together and discussed each predefined domain, the types of classroom behaviors and instructional practices to be documented, and the procedures for recording field notes. This process was undertaken to establish a shared understanding of the observation domains and recording procedures, thereby promoting consistency across observers. No formal observer-training program was conducted; rather, observer preparation consisted of this structured calibration process, supported by the observers’ prior experience in HPE research and qualitative classroom observation. Observers were not blinded to the instructional condition because the pedagogical modifications introduced in the redesigned sessions were directly observable.

Following each observation, the observers independently reviewed their field notes and subsequently compared and discussed their observations. Differences in observations or emphasis were discussed to develop a comprehensive integrated observation record for each lesson. These integrated observation reports constituted the observation dataset used in the subsequent qualitative analysis.

#### Development of the AI-supported educational intervention

Following the classroom observations, the existing learning objectives and teaching materials were analyzed using a purpose-built ChatGPT-based conversational support tool developed specifically for this study. The intervention was created in March 2026 using ChatGPT Plus (GPT-5.4 Thinking).

A single master prompt, rather than lesson-specific prompts, was developed to provide a standardized instructional design framework that could be adapted to different lesson contents. The master prompt was designed based on learner-centered and outcome-/competency-based educational principles, emphasizing constructive alignment between learning outcomes, instructional strategies, and assessment, together with an eight-step educational transformation algorithm developed by the research team REF (Figure 1). Its final version was established through iterative pilot testing and successive revisions to ensure clarity, consistency, and applicability across different educational contexts. The complete master prompt is provided in Supplementary Material 2.

**Figure 1.**
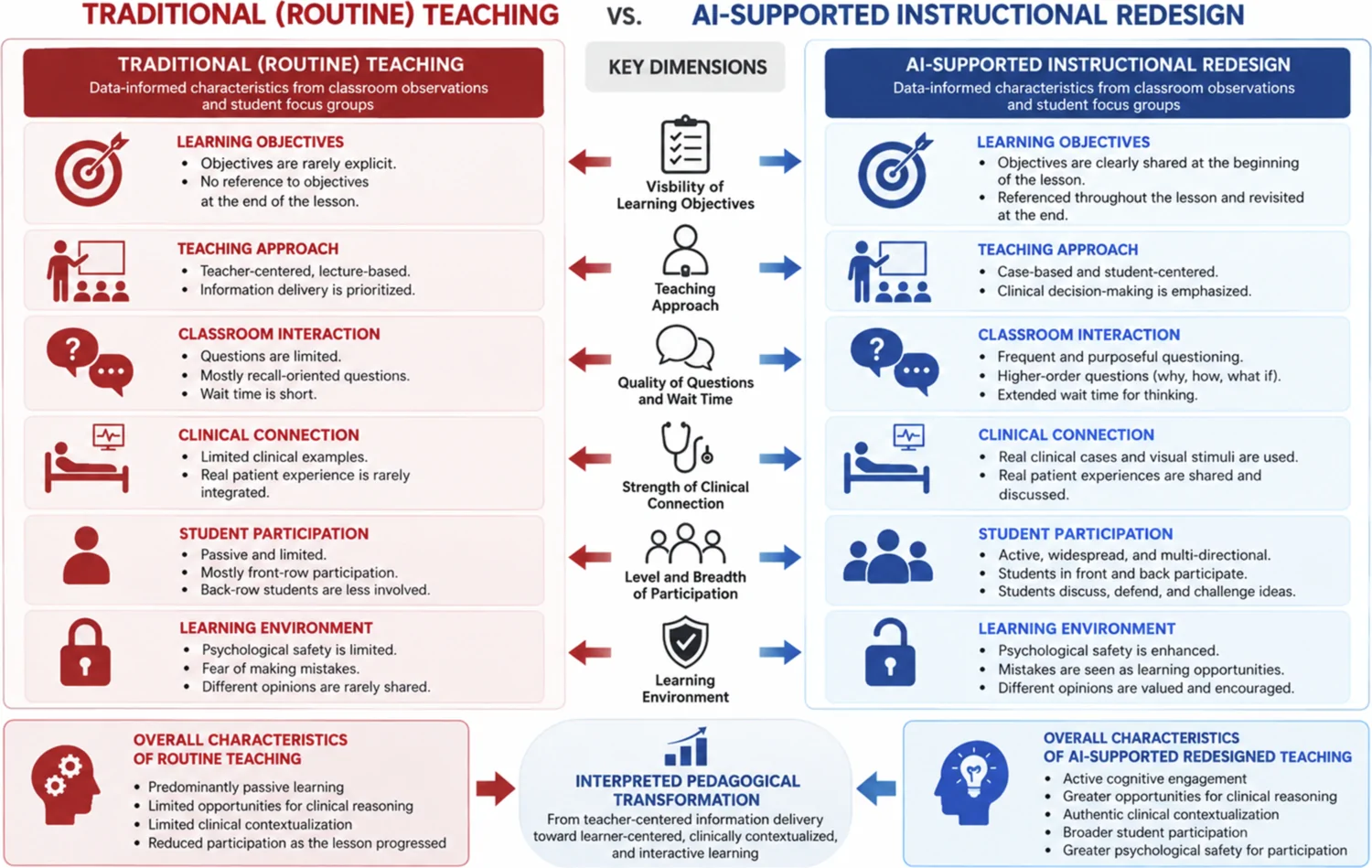
Provisional data-informed conceptual model of AI-supported pedagogical transformation in undergraduate dental education.

The ChatGPT-supported tool was designed to assist educators in enhancing the clinical relevance of their teaching by generating recommendations to (1) align learning objectives with intended clinical competencies, (2) propose authentic clinical scenarios and patient cases that explicitly linked theoretical concepts with clinical practice, (3) enrich classroom discussions through reflective and clinically oriented questioning strategies, and (4) strengthen opportunities for active student participation through learner-centred instructional activities.

For each lesson, the existing lesson material was entered into the same master prompt, and the generated recommendations were reviewed collaboratively by the research team and the respective lesson instructor. The initial AI-generated output was treated as instructional design input rather than as a finalized lesson plan and was subsequently reviewed, selected, and adapted by the research team and instructor as needed. The final instructional plans were developed through discussion and consensus, ensuring that all proposed modifications were pedagogically appropriate, aligned with the intended learning outcomes, and feasible within the existing curriculum while preserving the original lesson objectives. The recommendations that were considered pedagogically inappropriate, insufficiently aligned with the intended learning outcomes, or infeasible within the existing curricular context were not incorporated into the final instructional plans. AI-generated recommendations were therefore treated as suggested curricular designs rather than being automatically incorporated into the lessons, with instructors retaining responsibility for their selection, adaptation, and final implementation. The number of conversational rounds and the individual recommendations that were accepted, modified, or rejected were not prospectively logged and therefore were not retrospectively quantified.

To minimize educator-related bias, the revised lesson plans were implemented by two faculty members from the same academic department who had comparable undergraduate teaching experience, had been responsible for fourth-year lessons within the same curriculum, and had both completed formal faculty development training in health professions education. These similarities were considered sufficient to provide a comparable educational background while allowing implementation by different instructors. Following completion of the redesigned lesson plans, both instructors were given two weeks to familiarize themselves with the recommendations and adapt their teaching materials before delivering the revised sessions.

These instructors were provided with two weeks to review the ChatGPT-generated recommendations and adapt their teaching materials before delivering the revised sessions.

#### Students’ focus group discussions

Following the completion of each enhanced lesson, semi-structured focus group duscission (FGDs) were conducted with students who had attended both the conventional and the AI-supported sessions. Following an announcement after each lesson, eligible students were invited to volunteer for the FGDs. For the Atraumatic Restorative Treatment lesson, 16 students volunteered, and the first 10 volunteers were invited to participate; all 10 attended. For the Abscess Management and Gangrene in Children lesson, 10 students volunteered and were invited, of whom 8 attended. Thus, two focus groups were conducted with a total of 18 students. The Atraumatic Restorative Treatment focus group comprised six female and four male students, whereas the Abscess Management and Gangrene in Children focus group comprised four female and four male students. The discussions explored participants' perceptions of the clinical relevance of the lesson, the contribution of the ChatGPT-supported instructional modifications to learning, and the visibility of connections between theoretical concepts and clinical practice. When data saturation was reached within each lesson-specific focus group, no additional FGDs were conducted.

Both FDGs were conducted in a quiet meeting room within the Faculty of Dentistry to provide a comfortable environment for open discussion. Each discussion lasted approximately 60–75 minutes and was facilitated by a member of the research team using a semi-structured discussion guide (Supplementary Material 3). Both focus groups were facilitated by the same researcher (SA), an experienced medical education researcher with substantial experience in conducting qualitative research in dental, medical, and other health professions education. The facilitator had no teaching, assessment, or supervisory role in relation to the participating students. The facilitator was therefore not involved in the participants’ academic evaluation or progression. All discussions were audio-recorded with participants' written informed consent and were transcribed verbatim for analysis. Field notes were also taken during each session to capture contextual observations and non-verbal interactions that supported interpretation of the discussion data.

### Data analysis

All discussions were audio-recorded, transcribed verbatim, and analyzed using Braun and Clarke's six-phase reflexive thematic analysis approach [18]. Within the interpretivist orientation of the study, coding was primarily inductive and data-driven.

Theme development was iterative rather than predetermined. During successive rounds of analysis, candidate themes were combined, separated, or refined according to the coherence and distinctiveness of the patterns of meaning they captured. The final thematic structure was determined when the themes provided a coherent and conceptually distinct representation of the patterns identified across the dataset.

Observation notes and student focus groups were initially examined within their respective data sources and subsequently compared during theme development to achieve methodological triangulation and to enhance the depth of interpretations. Focus-group data primarily informed students’ experiences and perceptions, whereas observation data provided contextual evidence regarding instructional practices, classroom interactions, and student participation. Two researchers initially engaged with and coded the data independently to generate multiple interpretive perspectives rather than to assess inter-rater reliability. Their interpretations were subsequently examined through reflexive discussion, with areas of convergence and divergence used to refine the developing themes rather than consensus being treated as a criterion of analytic quality.

### Trustworthiness

The rigor of the study was enhanced using Lincoln and Guba's criteria for qualitative research [19]. Credibility was strengthened through methodological triangulation across classroom observations and student focus groups. Dependability was supported by maintaining a detailed audit trail throughout data collection and analysis. Confirmability was supported through reflexive researcher discussions, consideration of alternative interpretations, and documentation of analytic decisions, while transferability was facilitated by providing rich descriptions of the educational context and intervention.

### Researcher reflexivity

The researchers approached this study from an educational innovation perspective and were actively involved in designing the AI-supported instructional framework. Recognizing that this involvement could influence data interpretation, particular attention was paid to maintaining reflexivity throughout the research process. Data from classroom observations and students’ focus groups were triangulated, and emerging interpretations were discussed among the research team until consensus was achieved. Throughout the analysis, the researchers sought to distinguish participants' experiences from their own expectations regarding the educational potential of AI-supported instructional redesign.

### Ethical considerations

Ethical approval was obtained from the Scientific Research Ethics Committee of Karadeniz Technical University Faculty of Dentistry (Approval No. 2026/8, Date: 08.01.2026). Written informed consent was obtained from all participants prior to data collection of focus groups discussions. The study was conducted in accordance with the principles of the Declaration of Helsinki.

The clinical photographs used in the redesigned sessions were pre-existing materials obtained by the instructors during clinical practice, for which patient consent for educational use had been obtained.

## Results

The integrated analysis revealed that the impact of ChatGPT was not reflected primarily in the introduction of new technological features, but in the redesign of pedagogical processes through which clinical knowledge was taught, discussed, and applied. Across observations and focus groups, participants consistently described a shift from teacher-led information delivery toward interactive, clinically contextualized learning that actively engaged students in clinical reasoning, collaborative discussion, and professional meaning-making. To enhance analytic transparency, Table 1 presents the pathway from the eight interpretive subthemes to the four overarching themes and illustrates how classroom observation and focus-group data contributed to, converged within, or qualified each theme.

**Table 1.** Analytic pathway and integration of classroom observation and focus-group data across themes.

Final theme	Interpretive subthemes	Classroom observation evidence	Focus-group evidence	Integration of sources
1. From teacher-centered knowledge delivery to facilitation of clinical reasoning	1. Shift from information delivery to questioning and reasoning 2. Making and justifying clinical decisions	Baseline sessions were dominated by information delivery and factual questioning; redesigned sessions included interpretation of images, comparison of alternatives, justification of decisions, and discussion among students.	Students described moving from listening to actively thinking, diagnosing, recalling relevant information, and deciding what they would do clinically.	**Convergence:** Both sources indicated a shift from passive receipt of information toward active clinical reasoning.
2. From passive attendance to active cognitive engagement	3. Sustained attention and cognitive involvement 4. Participation through interactive learning activities	Baseline observations showed declining attention, high information density, and limited participation. Redesigned sessions included polling, visual prompts, audience-response activities, and small-group discussion with broader participation.	Students reported sustained attention and continued cognitive involvement, including monitoring whether their reasoning and responses were correct.	**Convergence:** Observed increases in participation corresponded with students’ reported cognitive engagement.
3. Building clinical meaning through authentic context and collaborative dialogue	5. Authentic clinical contextualization 6. Collaborative construction of clinical meaning	Redesigned sessions used clinical images, real patient examples, visual diagnosis, clinical scenarios, and discussion of instructors’ clinical experiences.	Students described real cases and visual examples as making concepts clearer, more memorable, and easier to connect with future clinical encounters.	**Convergence:** Both sources supported the role of authentic clinical contexts in connecting theoretical knowledge with clinical practice.
4. Creating educational climate for professional learning through psychological safety	7. Encouragement of alternative viewpoints and reasoning 8. Participation without immediate judgment	Observations showed discussion of different opinions and incorrect answers, with instructors asking students to explain reasoning rather than immediately evaluating responses.	Students reported that reduced fear of judgment facilitated participation, although speaking or making mistakes in front of a large class remained stressful.	**Convergence with qualification:** Both sources supported a more open participatory climate, while focus-group data additionally identified persistent anxiety related to large-group participation.

### From information transmission to clinical meaning-making: how AI-augmented instructional design reshaped learning

Analysis of classroom observations conducted before and after AI-supported instructional redesign, together with students' FGDs, revealed that the transformation extended far beyond the introduction of a new technology. Rather than changing the content of teaching, ChatGPT-supported instructional design altered the pedagogical processes through which students engaged with clinical knowledge.

The analysis followed 6-step model of Braun and Clark thematic analysis framework. Initial coding generated numerous descriptive codes that were progressively clustered into eight interpretive subthemes. Through iterative comparison across classroom observations and focus group discussions, these subthemes were organized into four overarching themes describing the pedagogical transformation associated with AI-supported instructional redesign (Figure 2).

**Figure 2.**
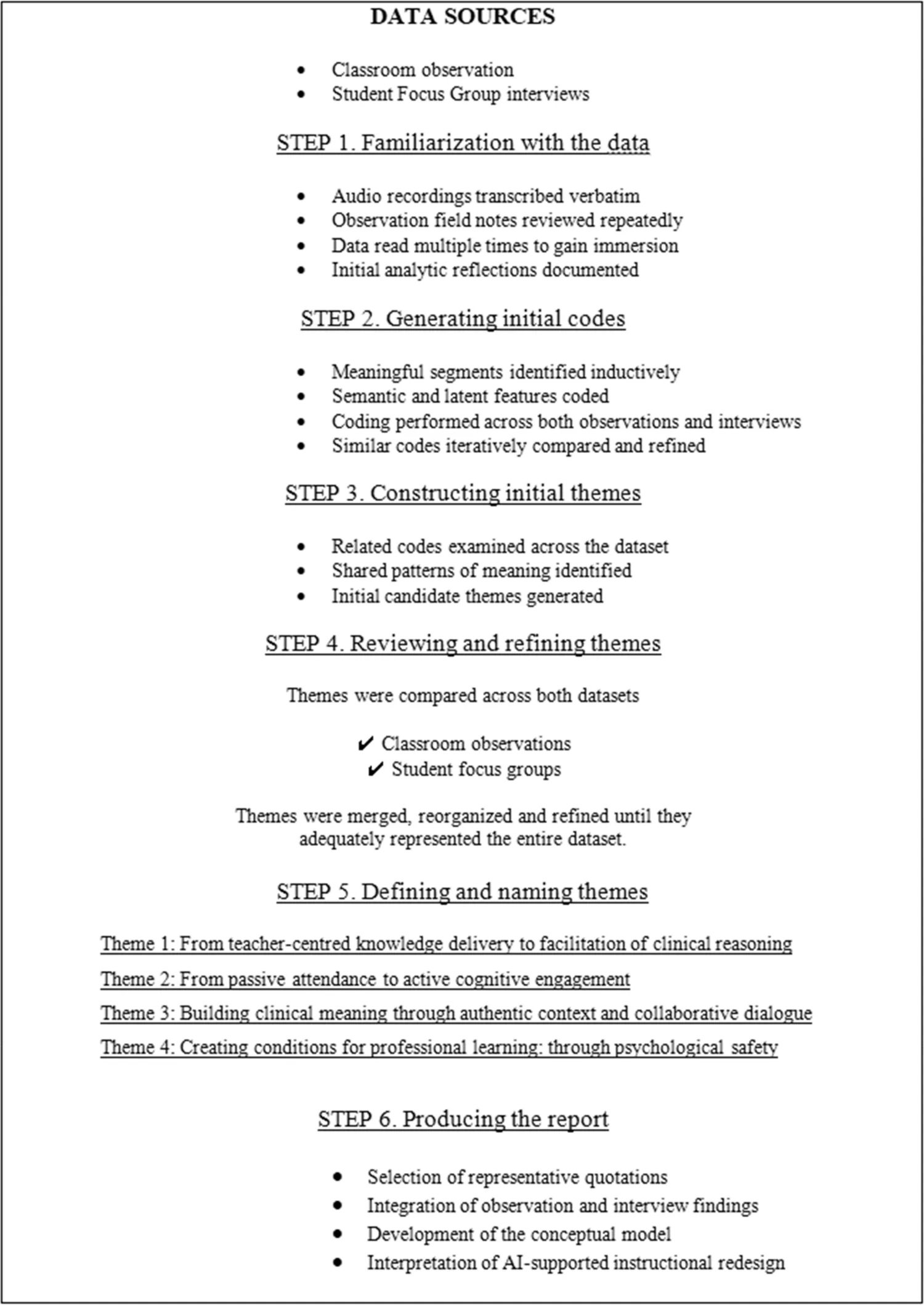
Six-step reflexive thematic analysis process following Braun and Clarke.

From our inductive thematic analysis, four interconnected themes emerged that explained the phenomenon of the transformation of the educational redesign.

**Theme 1. From teacher-centered knowledge delivery to facilitation of clinical reasoning**

Across the baseline classroom observations, teaching was predominantly organized around information delivery. Although instructors occasionally posed questions, these questions were generally answered by the instructor shortly afterward or focused on factual recall. Learning objectives were rarely made explicit, clinical reasoning remained largely implicit, and students participated primarily as listeners.

Field notes repeatedly documented that instructors dominated classroom discourse, while students had limited opportunities to construct explanations or justify clinical decisions. Questions typically assessed memory rather than reasoning, and little time was provided for learners to elaborate their thinking.

One field note stated:

*“Questions were asked, but the instructor answered them immediately before students had sufficient time to think.”*

Similarly,

*“Learning objectives were not explicitly introduced at the beginning of the lesson.”*

The AI-supported lessons demonstrated a markedly different pedagogical structure.

Rather than explaining clinical concepts directly, instructors continuously invited students to interpret clinical images, justify diagnostic decisions, compare alternative answers, and defend their reasoning.

Observation notes frequently documented statements such as:

*“Why did you choose this image?”*

*“Different answers were discussed.”*

*“Students debated their decisions.”*

*“The instructor encouraged discussion among students rather than immediately providing the correct answer.”*

These observational findings closely aligned with students' own descriptions of the learning process.

Students explained that they were no longer merely receiving information but were continuously required to make diagnostic decisions.

For example, one participant stated:

*“The instructor kept asking questions, so we had to remember the patient's age, photographs, and previous information. We stayed engaged throughout the lesson.”*

Another explained:

*“It wasn't just listening anymore. We had to think, diagnose, and decide what we would do.”*

**Theme 2. From passive attendance to active cognitive engagement**

Baseline observations consistently suggested that students became increasingly passive as lessons progressed.

Observers noted decreasing attention after approximately thirty minutes, rapid instructor pacing, extensive information density, and limited opportunities for participation.

One observation recorded:

*“Student motivation and attention noticeably decreased after approximately thirty minutes.”*

Another noted:

*“Information density was very high.”*

During AI-supported sessions, however, student behavior changed substantially.

Visual prompts, audience-response activities, clinical polling, and small-group discussions repeatedly interrupted passive listening.

Field notes documented:

*“Almost all students responded.”*

*“Students immediately reached for their phones to participate in polling.”*

*“Even students sitting in the back of the classroom became involved.”*

*“Students discussed possible answers within their groups before responding.”*

The focus groups confirmed these observations.

Students repeatedly described remaining cognitively engaged throughout the session.

One student explained:

*“Normally I lose my attention after five or ten minutes. In this lesson I didn't even look at my phone.”*

Another participant reflected:

*“After answering the first question, I kept wondering whether my reasoning was correct, so I followed the whole lesson.”*

**Theme 3. Building clinical meaning through authentic context and collaborative dialogue**

One of the glaring differences between the two instructional approaches was about the role of authentic clinical context.

Baseline observations suggested that clinical examples were relatively limited and often served only to illustrate previously presented information.

In contrast, AI-supported lessons were organized around authentic clinical scenarios, visual diagnosis, and collaborative interpretation.

Observation notes repeatedly highlighted:

*“Clinical images stimulated discussion.”*

*“Real clinical photographs attracted the attention of the entire classroom.”*

Instructors also incorporated their own clinical experiences as discussion triggers rather than as isolated anecdotes.

Students perceived these contextual changes as highly meaningful.

One participant stated:

*“Our instructor showed a real patient that we had actually seen in the clinic. That made everything much clearer.”*

Another explained:

*“Visual cases stay in my mind. When I encounter something similar in the clinic, I remember the lesson.”*

**Theme 4. Creating educational climate for professional learning through psychological safety**

Observational data indicated that instructors increasingly encouraged students to express alternative viewpoints and discuss incorrect answers without immediate evaluation.

Instead of emphasizing correctness, instructors frequently redirected discussion by asking learners to explain their reasoning.

Observation notes included comments such as:

*“Different opinions were openly discussed.”*

*“The instructor asked students why they chose their answers.”*

Students consistently interpreted this instructional climate as psychologically safer.

One participant explained:

*“If the instructor had simply asked us to raise our hands, I would have felt uncomfortable.”*

Another stated:

*“Being able to answer without feeling judged helped me participate.”*

Nevertheless, students also emphasized that speaking in front of large groups remained emotionally challenging.

As one participant observed:

*“Making a mistake in front of 120 classmates still feels stressful.”*

### Conceptual synthesis of the findings: the integrative model

To integrate the findings across observations and focus group discussions, we developed a conceptual model that illustrates the pedagogical transformation associated with AI-supported instructional redesign (Figure 3). Instead of depicting ChatGPT as a direct instructional tool, the model synthesizes how AI-assisted lesson planning reshaped teaching practices and learning processes. The observations demonstrated changes in instructional design and classroom interactions, while the focus groups revealed how students experienced these changes in terms of cognitive engagement, clinical reasoning, and professional learning. Collectively, our data suggests that ChatGPT functioned as an instructional design partner, enabling a shift from teacher-centered information delivery toward learner-centered, clinically contextualized education. The integrative model summarizes the sequential relationships identified across the data and provides an explanatory framework for understanding how AI-supported instructional redesign facilitated meaningful learning in undergraduate dental education.

**Figure 3.**
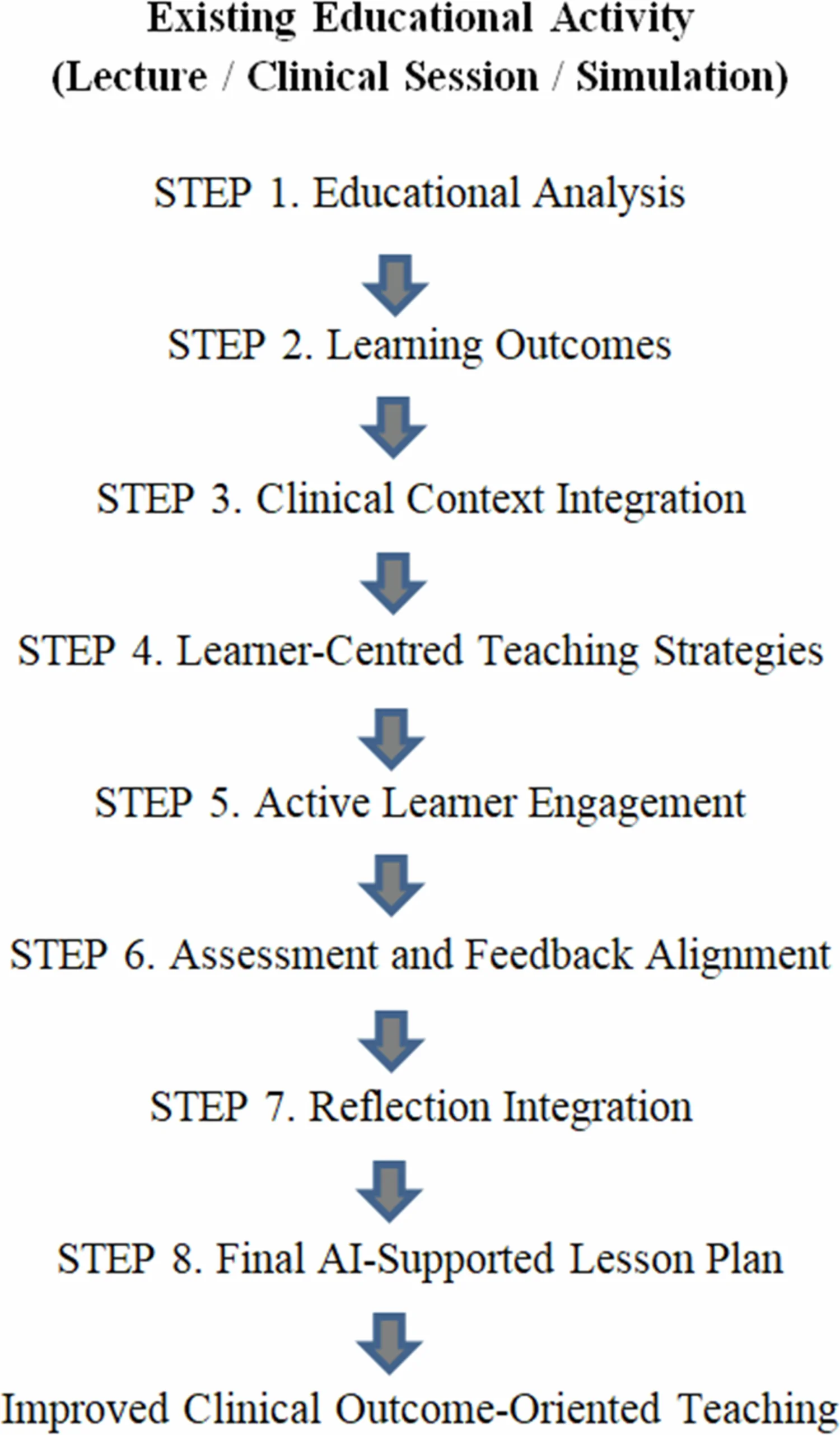
Eight-Step AI-Supported instructional redesign framework.

## Discussion

The present study highlights a significant role of the AI-supported instructional redesign to enhance undergraduate dental lessons for preclinical subjects. This educational intervention can offer support to medical educators to design and facilitate learning, rather than by replacing teaching or directly interacting with students. In our work, faculty used AI-generated recommendations to strengthen the alignment between learning objectives, authentic clinical scenarios, classroom activities, and expected clinical outcomes. Students experienced the redesigned sessions as promoting clinical reasoning, cognitive engagement, meaningful learning, and clinical relevance. These findings are consistent with the emerging corpus of evidence that the educational value of GenAI essentially lies in augmenting instructional design and in providing cognitive support while preserving educators' central pedagogical role [20–22]. However, these changes cannot be attributed to AI independently of the pedagogical strategies implemented through the redesign. ChatGPT functioned as a planning and design-support tool that helped educators operationalize learner-centred, clinically contextualized, and active learning strategies. The findings should therefore be interpreted as reflecting the educational value of AI-supported instructional redesign rather than evidence of superior learning outcomes attributable to AI itself.

An important finding, in our study, was that the educational value of active learning appeared to arise from engaging students in explicit clinical reasoning rather than simply increasing classroom participation. Students consistently described learning activities that required them to justify diagnoses, defend treatment decisions, evaluate alternative explanations, and explain the rationale underlying their clinical judgments. This finding indicates that meaningful active learning depends not merely on participation, but on the cognitive demands embedded within instructional tasks. This interpretation aligns with recent evidence showing that problem-based learning, case-based learning, team-based learning, and other active learning approaches improve clinical reasoning by requiring learners to generate hypotheses, integrate evidence, and justify decisions [23,24]. Similar mechanisms have been reported in Learning-by-Concordance, AI-supported case-based learning, and flipped classroom, where educational benefit emerges through structured reasoning, reflection, and iterative refinement of clinical decisions [25,26]. Our findings reinforce this literature by highlighting explicit articulation and revision of clinical reasoning through dialogue as a central feature of cognitively active learning.

Our study findings reinforce the principles of outcome-based and competency-based education by demonstrating that AI is most valuable when integrated within coherent instructional design rather than implemented as an isolated educational technology. In the present study, AI-supported recommendations aligned learning objectives, classroom activities, authentic clinical discussions, and expected competencies, thereby strengthening the clinical relevance of preclinical teaching. Faculty members redesigned existing lessons by incorporating realistic patient scenarios, clinical images, and case discussions that enabled students to connect theoretical concepts with authentic clinical practice. These findings are consistent with recent literature emphasizing that AI integration should support curriculum alignment, competency frameworks, and authentic assessment rather than technology-driven innovation alone [26–29]. They also align with situated learning theory, which proposes that meaningful learning occurs when knowledge is constructed within authentic professional contexts resembling future clinical practice [30–32]. Collectively, these findings suggest that AI-supported instructional redesign enhances learning not merely by introducing clinical examples but by embedding knowledge within authentic clinical contexts that facilitate transfer to professional practice.

Beyond strengthening clinical reasoning, the redesigned learning environment also promoted deeper cognitive engagement and psychological safety. Students described remaining mentally engaged throughout lessons because they were expected to predict, question, justify, and discuss clinical decisions while feeling comfortable expressing alternative opinions and making mistakes without fear of judgment. These experiences are consistent with the evidence that AI-supported instructional environments enhance learning when they facilitate higher-order thinking, collaborative dialogue, and active knowledge construction rather than simple content delivery [33,34]. Similarly, psychologically safe learning environments have been associated with greater learner engagement and meaningful participation, particularly when educators facilitate interaction instead of allowing technology to replace human teaching [35–37].

Finally, an important contribution of this study is the observation that the primary educational impact of AI occurred at the educator level rather than through direct student use. This finding supports recent calls for faculty development initiatives that position AI as a pedagogically informed design partner rather than a content generator [38]. Consequently, AI-supported instructional redesign may represent a practical and scalable approach for supporting sustainable curriculum innovation in health professions education.

## Limitations

This study has several limitations. First, it was conducted at a single institution and involved two fourth-year preclinical dental lessons delivered by two faculty members, which may limit the transferability of the findings to other educational contexts. Second, although students provided rich insights into their learning experiences, the study did not include discussions with the participating faculty members. Consequently, educators' perspectives on the feasibility, usability, and instructional value of the AI-supported redesign process were not explored.

## Future research

Future research should examine the implementation of AI-supported instructional design across multiple institutions, dental disciplines, and larger groups of educators to enhance the transferability of the findings. Studies incorporating faculty perspectives, longitudinal evaluation of educational outcomes, and objective measures of students' clinical reasoning and transfer of learning would provide a more comprehensive understanding of the educational impact of AI-supported curriculum enhancement.

## Conclusion

This study showcases scalable data about the potential role of AI-supported instructional design which can function as an effective pedagogical design partner, enabling educators to modify existing dental lessons for stronger clinical engagements by the learners. Instead of replacing educators or directly influencing student learning, AI-augmented changes in instructional design that transformed classroom teaching practices. The pedagogical redesign, in our study, was reflected in students' experiences as deeper clinical reasoning, greater cognitive engagement, more meaningful learning, and stronger perceptions of the clinical relevance of preclinical education. These findings suggest that AI-supported instructional redesign represents a practical and scalable strategy for advancing outcome-oriented health professions education while preserving the central role of educators in facilitating learning.

## Supplementary Material

Supplementary Material 1_Revised.docx Supplementary Material 1_Revised.docx

Supplementary Material 2_Revised.docx Supplementary Material 2_Revised.docx

Supplementary_Material_3_Revised clean.docx Supplementary_Material_3_Revised clean.docx

## Data Availability

The data that support the findings of this study are available from the corresponding author, upon reasonable request.

## References

[1] Nafea ET , Alhussin AM , Alkuhayli SA , et al. Shaping professional identity in dentistry: a cross-sectional study of value prioritization among undergraduate students. BMC Med Educ. 2025;25(1):1613. doi: 10.1186/s12909-025-08209-0 PMC12625722 41250125

[2] Serrano CM , Botelho MG , Wesselink PR , et al. Challenges in the transition to clinical training in dentistry: An ADEE special interest group initial report. Eur J Dent Educ: Official Journal of the Association for Dental Education in Europe. 2018;22(3):e451–e457. doi: 10.1111/eje.12324 29396888

[3] Elvén M , Welin E , Wiegleb Edström D , et al. Clinical Reasoning Curricula in Health Professions Education: A Scoping Review. J Med Educ Curric Dev. 2023;10:23821205231209093. doi: 10.1177/23821205231209093 PMC10605682 37900617

[4] Harden RM . Outcome-based education: The future is today. Med Teach. 2007;29(7):625–629. doi: 10.1080/01421590701729930 18236247

[5] Gruppen LD . Outcome-based medical education: implications, opportunities, and challenges. Korean J Med Educ. 2012;24(4):281–285. doi: 10.3946/kjme.2012.24.4.281 PMC8813364 25813324

[6] Eysenbach G . The role of ChatGPT in medical education. J Med Internet Res. 2023;25:e46885. 10.2196/46885 PMC10028514 36863937

[7] Kung TH , Cheatham M , Medenilla A , et al. Performance of ChatGPT on USMLE. PLOS Digital Health. 2023;2(2):e0000198. doi: 10.1371/journal.pdig.0000198 PMC9931230 36812645

[8] Wu Y , Zheng Y , Feng B , et al. Embracing ChatGPT for Medical Education: Exploring Its Impact on Doctors and Medical Students. JMIR Med Educ. 2024;10:e52483. doi: 10.2196/52483 PMC11043925 38598263

[9] Kavadella A , Dias da Silva MA , Kaklamanos EG , et al. Evaluation of ChatGPT's Real-Life Implementation in Undergraduate Dental Education: Mixed Methods Study. JMIR Med Educ. 2024;10:e51344. doi: 10.2196/51344 PMC10867750 38111256

[10] Uribe SE , Maldupa I , Schwendicke F . Integrating Generative AI in Dental Education: A Scoping Review of Current Practices and Recommendations. Eur J Dent Educ: Official Journal of the Association for Dental Education in Europe. 2025;29(2):341–355. doi: 10.1111/eje.13074 PMC12006694 39891376

[11] Yazdi NA , Zamaniahari U , KhadiviMaleki H , et al. Readiness to use artificial intelligence: a comparative study among dental faculty members and students. BMC Med Educ. 2025;25(1):1006. doi: 10.1186/s12909-025-07621-w PMC12228260 40615869

[12] Chau RCW , Thu KM , Yu OY , et al. Performance of generative artificial intelligence in dental licensing examinations. Int Dent J. 2024;74(3):616–621. doi: 10.1016/j.identj.2023.12.007 PMC11123518 38242810

[13] Chau RCW , Thu KM , Yu OY , et al. Evaluation of chatbot responses to text-based multiple-choice questions in prosthodontic and restorative dentistry. Dentistry Journal. 2025;13(7):279. doi: 10.3390/dj13070279 PMC12293279 40710124

[14] Fukuda H , Morishita M , Takahashi O , et al. Evaluation of the performance of ChatGPT-4o on oral surgery-related questions in the Japanese National Dental Examination. Int J Oral Maxillofac Surg. 2026;S0901-5027(26):00363–2. doi: 10.1016/j.ijom.2026.08.026 42680602

[15] Rokhshad R , Zhang P , Mohammad-Rahimi H , et al. Accuracy and consistency of chatbots versus clinicians for answering pediatric dentistry questions: A pilot study. J Dent. 2024;144:104938. doi: 10.1016/j.jdent.2024.104938 38499280

[16] Creswell JW , Poth CN . Qualitative inquiry and research design. 4th ed. Sage; 2018.

[17] Yin RK . Case study research and applications: Design and methods. 6th ed. Sage Publications; 2018.

[18] Braun V , Clarke V . Using thematic analysis in psychology. Qual Res Psychol. 2006;3(2):77–101. doi: 10.1191/1478088706qp063oa

[19] Nowell LS , Norris JM , White DE , et al. Thematic Analysis: Striving to Meet the Trustworthiness Criteria. Int J Qual Methods. 2017;16(1):1–13. doi: 10.1177/1609406917733847

[20] Kibar PN , Ilgaz H . The intersection of artificial intelligence and instructional design practice: a systematic review. Education Tech Research Dev. 2026;74:1989–2015. doi: 10.1007/s11423-026-10624-z

[21] Kozan K , Hur J , Kim I , et al. Instructional Designers’ Integration of Generative Artificial Intelligence into Their Professional Practice. Educ Sci. 2025;15(9):1133. doi: 10.3390/educsci15091133

[22] Suresh D , Dilek M , Karabulut-Ilgu A , et al. Generative AI in Learning Design Practice: Building an Online Course for Biomedical Sciences Research Programs. International Journal of Designs for Learning. 2025;16(2):244–265. doi: 10.14434/ijdl.v16i2.41995 PMC12965148 41799159

[23] Salari B , Amini M . The impact of different teaching methods on clinical reasoning and clinical decision-making of dentistry students: A systematic review. AADS Proceedings. 2026;90(1):33–42. doi: 10.1002/jdd.13930 40344237

[24] Zhao L , He J , Han Z , et al. Impact of problem-based learning on the development of clinical reasoning skills in esophageal cancer teaching. Medicine (Baltimore). 2026 Feb 6;105(6):e47451. doi: 10.1097/MD.0000000000047451 PMC12885694 41650043

[25] Newsom LC . Learning-by-concordance to enhance clinical reasoning skills in student pharmacists. Am J Pharm Educ. 2026;90(4):101963. doi: 10.1016/j.ajpe.2026.101963 41802544

[26] Wang Y , Zhou M , Meng H . Artificial intelligence for teaching, training, and assessment in dental education: a domain-based scoping review. Front Med (Lausanne). 2026 Apr 1;13:1775853. doi: 10.3389/fmed.2026.1775853 PMC13078982 41994464

[27] Ali K . Rethinking the Integration of AI in Learning and Assessment in Health Professions Education. Eur J Dent Educ. 2026;30(3):776–778. doi: 10.1111/eje.70144 PMC13383364 41839807

[28] El-Hakim M , Anthonappa R , Fawzy A . Artificial Intelligence in Dental Education: A Scoping Review of Applications, Challenges, and Gaps. Dent J. 2025;13(9):384. doi: 10.3390/dj13090384 PMC12468998 41002657

[29] Osathanon T , Vacharaksa A , Schwendicke F , et al. Preparing the AI-Ready Dentist: A Call for a Competency Framework in Dental Education. Int Dent J. 2026;76(2):109450. doi: 10.1016/j.identj.2026.109450 PMC12933789 41719952

[30] Cho H , Jeong H , Yu J , et al. Becoming a doctor: Using social constructivism and situated learning to understand the clinical clerkship experiences of undergraduate medical students. BMC Med Educ. 2024;24:236. doi: 10.1186/s12909-024-05113-x PMC10916183 38443907

[31] Liljedahl M , Björck E , Bolander Laksov K . How workplace learning is put into practice: Contrasting the medical and nursing contexts from the perspective of teaching and learning regimes. Adv Health Sci Educ. 2023;28:811–826. doi: 10.1007/s10459-022-10195-7 PMC10356663 36459259

[32] Stoffels M , van der Burgt SME , Bronkhorst LH , et al. Learning in and across communities of practice: Health professions education students' learning from boundary crossing. Adv Health Sci Educ. 2022;27:1423–1441. doi: 10.1007/s10459-022-10135-5 PMC9274184 35819568

[33] Pang X , Zou J , Zhang X , et al. The impact of artificial intelligence-assisted teaching on medical students' learning outcomes: An integrated model based on the ARCS model and constructivist theory. BMC Med Educ. 2025;25:1309. doi: 10.1186/s12909-025-07826-z PMC12492703 41039317

[34] Pham TD , Karunaratne N , Exintaris B , et al. The impact of generative AI on health professional education: A systematic review in the context of student learning. Med Educ. 2025;59(12):1280–1289. doi: 10.1111/medu.15746 PMC12686775 40533396

[35] Duan S , Liu C , Rong T , et al. Integrating AI in medical education: A comprehensive study of medical students' attitudes, concerns, and behavioral intentions. BMC Med Educ. 2025;25:599. doi: 10.1186/s12909-025-07177-9 PMC12020173 40269824

[36] Sunmboye K , Strafford H , Noorestani S , et al. Exploring the influence of artificial intelligence integration on personalized learning: A cross-sectional study of undergraduate medical students in the United Kingdom. BMC Med Educ. 2025;25:570. doi: 10.1186/s12909-025-07084-z PMC12007362 40247306

[37] Wang Y , Ji Y . How do they learn: Types and characteristics of medical and healthcare student engagement in a simulation-based learning environment. BMC Med Educ. 2021;21(1):1–13. doi: 10.1186/s12909-021-02858-7 PMC8349045 34362346

[38] Khamis N , Ungaretti T . From curiosity to competence: A structured faculty development model for AI integration in health professions education. Med Sci Educ. 2025;35(6):2693–2698. doi: 10.1007/s40670-025-02573-8 PMC12961079 41798345

