# Supplementary material for: Advancing clinical integration in undergraduate dental education through AI-enabled instructional design: a qualitative thematic analysis
Source: Med Educ Online. 2026 Oct 19;31(1):2750709. doi: 10.1080/10872981.2026.2750709 (PMC13686208; doi:10.1080/10872981.2026.2750709)
Supplement: Supplementary file 1 [file ZMEO_A_2750709_SM6183.docx]

**Supplementary Material 1.** Classroom Observation Guide

**A. General Information**

- **Type of educational activity:**
  ☐ Didactic lecture ☐ Clinical teaching ☐ Simulation ☐ Case-based discussion

☐ Other: __________

- **Discipline:**
  ☐ Medicine ☐ Dentistry
- **Educational level:**
  ☐ Preclinical ☐ Clinical ☐ Internship ☐ Residency
- **Observed instructor:** ___________________________
- **Date / Duration of observation:** __________________
- **Observer:** ___________________________

**B. Learning Outcomes and Instructional Alignment**

| **Observation Criterion** | **No (0)** | **Partially (1)** | **Yes (2)** |
| --- | --- | --- | --- |
| Learning outcomes were clearly stated at the beginning of the session. | ☐ | ☐ | ☐ |
| Learning outcomes focused on what learners were expected to achieve. | ☐ | ☐ | ☐ |
| The instructional process was aligned with the stated learning outcomes. | ☐ | ☐ | ☐ |

**Observation Notes:**

**C. Learner Engagement and Interaction**

| **Observation Criterion** | **No (0)** | **Partially (1)** | **Yes (2)** |
| --- | --- | --- | --- |
| Learners actively participated in the educational process. | ☐ | ☐ | ☐ |
| Learners were given opportunities to ask questions and express their opinions. | ☐ | ☐ | ☐ |
| Interaction involved multiple learners rather than only individual exchanges. | ☐ | ☐ | ☐ |
| Learners interacted with their peers during the session. | ☐ | ☐ | ☐ |

**Observation Notes:**

**D. Instructor's Role**

| **Observation Criterion** | **No (0)** | **Partially (1)** | **Yes (2)** |
| --- | --- | --- | --- |
| The instructor primarily acted as a facilitator rather than solely as a knowledge provider. | ☐ | ☐ | ☐ |
| The instructor used open-ended questions to stimulate thinking. | ☐ | ☐ | ☐ |
| The instructor made learners' thinking processes visible during the session. | ☐ | ☐ | ☐ |
| Learners were given responsibilities appropriate to their level of training. | ☐ | ☐ | ☐ |

**Observation Notes:**

**E. Teaching Methods and Learning Activities**

| **Observation Criterion** | **No (0)** | **Partially (1)** | **Yes (2)** |
| --- | --- | --- | --- |
| Learner-centered instructional methods (e.g., case-based learning, small-group activities) were used. | ☐ | ☐ | ☐ |
| Learning activities were explicitly linked to authentic clinical contexts. | ☐ | ☐ | ☐ |
| Learners participated in clinical decision-making and/or problem-solving processes. | ☐ | ☐ | ☐ |

**Observation Notes:**

**F. Feedback and Assessment**

| **Observation Criterion** | **No (0)** | **Partially (1)** | **Yes (2)** |
| --- | --- | --- | --- |
| The instructor provided feedback to learners. | ☐ | ☐ | ☐ |
| Feedback was specific and constructive. | ☐ | ☐ | ☐ |
| Assessment activities were used to support learning. | ☐ | ☐ | ☐ |

**Observation Notes:**

**G. Reflection and Session Closure**

| **Observation Criterion** | **No (0)** | **Partially (1)** | **Yes (2)** |
| --- | --- | --- | --- |
| Learners were encouraged to reflect on their learning experiences. | ☐ | ☐ | ☐ |
| The session concluded by explicitly linking activities back to the intended learning outcomes. | ☐ | ☐ | ☐ |

**Observation Notes:**

**Strengths**

**Areas for Improvement**
