# Supplementary material for: Advancing clinical integration in undergraduate dental education through AI-enabled instructional design: a qualitative thematic analysis
Source: Med Educ Online. 2026 Oct 19;31(1):2750709. doi: 10.1080/10872981.2026.2750709 (PMC13686208; doi:10.1080/10872981.2026.2750709)
Supplement: Supplementary file 3 [file ZMEO_A_2750709_SM6185.docx]

**Supplementary Material 3.** Semi-structured focus group discussion guide.

**Introduction (Moderator)**

Thank you for participating in this discussion. We are interested in hearing about your experiences of the course. There are no right or wrong answers. Please feel free to share both positive and negative opinions. Everything discussed here will remain confidential.

**Opening Question**

**1. How would you describe your overall experience of this course?**

**Possible prompts**

- What aspects attracted your attention most?
- How did you feel during the lesson?

**Teaching–Learning Process**

**2. How would you describe the teaching approach used during this course?**

**Possible prompts**

- What teaching methods did you notice?
- How did the lesson differ from your usual classes?
- Were there any activities that particularly supported your learning?

**Clinical Relevance**

**3. How did the course help you connect theoretical knowledge with clinical practice?**

**Possible prompts**

- Were there moments when the clinical relevance became particularly clear?
- Which activities or examples helped establish these connections?
- Can you describe a specific example?

**Learner Engagement**

**4. How did the instructional approach influence your participation during the lesson?**

**Possible prompts**

- Asking questions
- Participating in discussions
- Sharing ideas
- Solving problems

**Clinical Thinking**

**5. Did the course influence the way you think about patient care or clinical decision-making? If so, in what ways?**

**Possible prompts**

- Clinical reasoning
- Diagnostic thinking
- Treatment planning
- Patient-centred care

**Comparison with Previous Learning Experiences**

**6. Compared with similar courses you have previously attended, what similarities or differences did you notice?**

**Possible prompts**

- Teaching methods
- Classroom interaction
- Clinical orientation
- Learning experience

**Perceived Impact of the Redesigned Lesson**

**7. Which aspects of the redesigned lesson contributed most to your learning, and why?**

**Possible prompts**

- Clinical cases
- Classroom discussions
- Instructor questions
- Interactive activities
- Reflection opportunities

**Suggestions**

**8. What suggestions would you make to further improve this type of instructional approach?**

**Possible prompts**

- What should definitely be retained?
- What could be improved?
- Would you like to see similar approaches used in other courses?

**Closing Question**

**9. Is there anything else you would like to add about your learning experience?**

**Moderator Notes (not asked verbatim)**

The moderator should:

- encourage participation from all students;
- use follow-up questions such as *"Could you tell me more about that?"*, *"Can you give an example?"*, or *"What makes you think so?"*;
- avoid leading questions or expressing agreement/disagreement;
- ensure balanced participation and avoid domination by individual participants.
